# Impact of Preoperative Filling-Phase Vesicoureteral Reflux on Febrile Urinary Tract Infections After Vesicostomy in Children

**DOI:** 10.7759/cureus.96856

**Published:** 2025-11-14

**Authors:** Shinsuke Yoshizawa, Kensuke Ohashi

**Affiliations:** 1 Department of Pediatric Urology, Saitama Children's Medical Center, Saitama, JPN

**Keywords:** febrile urinary tract infections, filling-phase vur, pediatric urology surgery, vesicostomy, vesicoureteral reflux

## Abstract

Introduction: Vesicostomy is a widely accepted urinary diversion procedure for pediatric patients with high-pressure bladders, such as those with vesicoureteral reflux (VUR) or neurogenic bladder (NB). This study aimed to investigate whether preoperative filling-phase VUR detected on voiding cystourethrography (VCUG) contributes to the recurrence of febrile urinary tract infections (fUTIs) following vesicostomy.

Materials and methods: This retrospective study analyzed 27 patients who underwent vesicostomy between January 2019 and December 2024. Patients were categorized according to the presence or absence of postoperative fUTIs. Clinical parameters included sex, presence of filling-phase VUR, bladder compliance, detrusor overactivity, posterior urethral valve (PUV), and intrarenal reflux (IRR). Statistical analysis was performed using the chi-squared test, with significance set at p<0.05.

Results: Of the 27 patients (21 males, six females), 17 (62.9%) developed fUTIs postoperatively. Filling-phase VUR was observed in 20 patients, 15 of whom developed fUTIs, while only two of seven patients without filling-phase VUR experienced fUTIs (p=0.03). The odds ratio (OR) for filling-phase VUR was 7.50. Other factors, including bladder compliance, detrusor overactivity, PUV, and IRR, showed no significant association.

Conclusion: Preoperative filling-phase VUR detected on VCUG is a significant risk factor for recurrent fUTIs following vesicostomy in children. Patients with filling-phase VUR may require closer follow-up and adjunctive management strategies to prevent postoperative infections.

## Introduction

Vesicostomy is one of the urinary diversion techniques commonly used in pediatric urology to manage voiding dysfunction due to high-pressure bladders, such as in cases of vesicoureteral reflux (VUR) or neurogenic bladder (NB) [1-4]. This approach can effectively reduce upper tract deterioration and febrile urinary tract infections (fUTIs) by providing a low-resistance outlet for urine. However, some patients still experience fUTIs even after vesicostomy, despite having adequate urine drainage and receiving continuous antibiotic prophylaxis (CAP). We hypothesized that among potential etiologies, the presence of filling-phase VUR may contribute to the persistent risk of fUTIs postoperatively. To explore this possibility, we conducted a retrospective cohort analysis at our institution.

## Materials and methods

This retrospective study included 27 pediatric patients who underwent vesicostomy at the Department of Pediatric Urology at Saitama Children's Medical Center in Saitama, Japan, between January 2019 and December 2024 (Table 1). The patients were divided into two groups based on the presence or absence of fUTIs after vesicostomy. fUTI was defined as ≥10⁵ colony-forming units/mL of a single pathogen in a catheterized or suprapubic aspirate specimen, accompanied by pyuria. Cases with multiple organisms or suspected contamination were excluded. The presence of a posterior urethral valve (PUV) was assessed using cystourethroscopy. Bacterial species isolated from positive cultures were recorded and summarized in the Results section. Table 2 shows the indications for vesicostomy.

**Table 1:** Patient background characteristics "+" indicates presence and "-" indicates absence. Compliance is expressed in mL/cmH_2_O. M: males; F: females; UTI: urinary tract infections; PUV: posterior urethral valve; DO: detrusor overactivity; CAP: continuous antibiotic prophylaxis; ST: sulfamethoxazole/trimethoprim; CCL: cefaclor; NB: neurogenic bladder; ARM: anorectal malformation; MMC: myelomeningocele; EA: esophageal atresia; MCDK: multicystic dysplastic kidney; AUV: anterior urethral valve; VACTERL: vertebral defects, anal atresia, cardiac defects, tracheo-esophageal fistula, renal anomalies, and limb abnormalities; IRR: intrarenal reflux; fUTI: febrile urinary tract infection; VUR: vesicoureteral reflux VUR grade was classified according to the International Reflux Grading System [5].

Patient no.	Sex	Birth weight (g)	VUR grade		Filling-phase VUR side	IRR side	PUV	Indication of vesicostomy	Operation age (month)	fUTI post- op	Bladder compliance (mL/H_2_O)	DO	CAP	Comorbidities
			Right	Left										
1	M	3088	Ⅳ	Ⅴ	Both	-	-	UTI	43	+	0.5	-	ST	ARM
2	M	2092	Ⅰ	Ⅳ	Both	-	-	NB due to MMC	15	+	4	-	-	MMC, right renal atrophy
3	M	3326	Ⅴ	Ⅴ	Both	-	+	UTI	2	+	-	-	-	Stoma relapse
4	F	2616	Ⅱ	Ⅴ	Both	Left	-	NB due to MMC	18	-	3.5	+	-	ARM, MMC, right renal atrophy
5	M	3087	Ⅳ	Ⅱ	Both	-	+	UTI	21	+	1	-	ST	Trisomy 21
6	F	2738	-	Ⅲ	Left	-	-	NB due to MMC	64	+	3.2	-	-	MMC
7	M	1709	Ⅴ	Ⅴ	Left	-	-	UTI	12	+	2.6	-	-	ARM, EA, dead
8	M	2570	-	Ⅲ	Left	-	-	UTI	203	-	3.6	-	-	
9	M	3114	Ⅴ	Ⅲ	Right	Right	+	PUV	5	-	9	-	-	VURD
10	M	3364	Ⅴ	Ⅳ	Both	Both	+	UTI	3	-	10	-	-	MMC
11	M	3636	Ⅴ	-	Right	-	AUV	Urinary retention	0	+	0.3	-	ST	AUV
12	M	3566	-	-	-	Left	-	UTI	240	+	30	+	-	Gaucher disease, NB
13	M	2665	Ⅴ	-	Left	Right	+	UTI	1	+	0.4	+	-	Left renal atrophy
14	M	3390	Ⅴ	Ⅲ	Right	-	+	UTI	0	+	25	-	ST	Candida nephritis
15	M	1904	-	-	-	-	Urethral atresia	Urinary retention	0	-	-	-	ST	Potter syndrome, right lenal dysplasia, left MCDK
16	M	3998	Ⅳ	Ⅳ	Left	-	-	UTI	4	+	7	-	ST	
17	F	2818	-	-	-	-	-	NB due to MMC	41	-	2.25	+	-	MMC
18	F	1012	Ⅱ	Ⅱ	Left	-	-	UTI	20	+	1.3	-	-	Bilateral ureteral ectopic opening, duplicate vagina
19	F	2745	Ⅱ	Ⅱ	Both	Both	-	UTI	3	+	59	-	-	VACTERL association
20	M	3010	Ⅳ	Ⅳ	-	-	+	UTI	3	-	4.6	-	ST	
21	M	3530	Ⅲ	Ⅱ	-	-	-	UTI	5	+	10	-	ST	ARM, bilateral atrophy
22	F	2860	Ⅳ	Ⅴ	Both	Both	-	UTI	18	+	9.4	-	ST	Trisomy 21
23	M	2030	-	-	-	-	-	Urinary retention	41	-	-	-	-	Trisomy 21, myelitis
24	M	2562	Ⅴ	Ⅴ	Both	Both	-	UTI	6	-	2.8	-	CCL	
25	M	3384	-	Ⅲ	-	-	-	NB due to MMC	44	-	-	-	-	MMC
26	M	2650	Ⅰ	Ⅳ	Left	Left	+	UTI	4	+	15	-	-	Left MCDK
27	M	2836	Ⅴ	Ⅴ	Both	Both	+	UTI	5	+	11.7	-	CCL	

**Table 2:** Indications for vesicostomy UTI: urinary tract infections

Indication	Number of patients (%)	Statistical test	P-value
Recurrent febrile UTI	18 (66.6%)	-	-
Neurogenic bladder (e.g., myelomeningocele)	5 (18.5%)	-	-
Obstructive uropathy (e.g., posterior urethral valve)	3 (11.1%)	-	-
Others/unknown	1 (3.8%)	-	-

The following clinical parameters were extracted from medical records and analyzed: sex, presence of filling-phase VUR on preoperative voiding cystourethrography (VCUG) (graded according to the International Reflux Grading System [5]), bladder compliance, detrusor overactivity (DO), PUV (in males only), and presence of intrarenal reflux (IRR). Urodynamic studies (UDS) were performed in 23 of the 27 patients to assess bladder function; bladder compliance ≤5 mL/cmH₂O was considered abnormal. We focused on preoperative filling-phase characteristics and did not analyze all possible urodynamic variables (e.g., maximum vesical pressure).

Statistical analyses were conducted using EZR version 1.54 (Jichi Medical University, Shimotsuke, Japan), a free graphical user interface for R. EZR is distributed under the GNU General Public License version 2 (GPL-2) and is widely used for medical statistics [6]. Post hoc power analysis was performed with G*Power 3.1 (Heinrich-Heine University, Düsseldorf, Germany), which is freeware for academic use [7]. A two-sided p-value of <0.05 was considered statistically significant. All software used (EZR, G*Power) were free and appropriately licensed for academic research use.

This study was approved by the Saitama Children's Medical Center Ethics Committee (approval number: 2024-06-008). Due to its retrospective design and the use of anonymized data, the requirement for informed consent was waived.

## Results

A total of 27 patients (21 males and six females) underwent vesicostomy during the study period. The median age at the time of surgery was five months. Among them, 17 patients (62.9%) developed fUTIs following vesicostomy.

The most common indication for vesicostomy was control of recurrent fUTIs (66.6%), followed by NB due to myelomeningocele (18.5%) and urinary retention from PUV or urethral obstruction (11.1%). Four patients (14.8%) were negative for VUR at the time of surgery.

Preoperative VCUG revealed filling-phase VUR in 20 patients, of whom 15 (75%) developed postoperative fUTIs. Among the seven patients without filling-phase VUR, only two (28.6%) developed fUTIs (p=0.03). The odds ratio (OR) for filling-phase VUR was 7.5, indicating a strong association.

UDS data were available in 23 patients. Of the 13 patients with low bladder compliance (<5 mL/cmH₂O), eight developed fUTIs, whereas among 10 patients with normal compliance, eight also experienced fUTIs. DO was observed in four patients, with two developing fUTIs; in contrast, 14 of 19 patients without DO developed fUTIs.

Among 11 patients with PUV, seven had postoperative fUTIs. In the IRR-positive group (n=10), six developed fUTIs, compared to 11 out of 17 in the IRR-negative group.

When stratified by age at surgery, 10 of 15 patients under one year of age developed fUTIs, compared to seven of 12 in those older than one year. This age difference was not statistically significant (OR=1.43).

Among all variables analyzed, only filling-phase VUR showed a statistically significant association with postoperative fUTIs (Table 3).

**Table 3:** Comparison of risk factors for postoperative fUTI after vesicostomy "+" indicates presence and "-" indicates absence. The χ^2^ test or Fisher's test was used as appropriate. Odds ratios and 95% confidence intervals were calculated using logistic regression. Post hoc power (for the association between filling-phase VUR and fUTI) was 0.78. Power analysis was performed with G*Power version 3.1 (effect size 0.55; α=0.05). All analyses were performed using EZR version 1.54. fUTI: febrile urinary tract infection; VUR: vesicoureteral reflux; PUV: posterior urethral valve; IRR: intrarenal reflux

Variable	Category	fUTI (+) (n (%))	fUTI (-) (n (%))	χ^2^/Fisher's p-value	Odds ratio (95% CI)
Filling-phase VUR	+/-	15 (75)/2 (28.6)	5 (25)/5 (71.4)	χ^2^=4.60; p=0.03	7.50 (1.09-51.52)
Sex	M/F	13 (65)/4 (66.7)	8 (35)/2 (33.3)	χ^2^=0.05; p=0.83	0.81 (0.11-6.09)
PUV	+/-	7 (63.6)/6 (66.7)	4 (36.4)/2 (33.3)	Fisher; p=0.6	1.05 (0.17-6.44)
Bladder compliance (mL/cmH_2_O)	<5/≧5	8 (61.5)/8 (80)	5 (38.5)/2 (20)	Fisher; p=0.34	0.40 (0.06-2.54)
Detrusor overactivity	+/-	2 (50)/14 (73.6)	2 (50)/5 (26.3)	Fisher; p=0.35	0.36 (0.04-3.26)
Operation age	<1 year/≧1 year	10 (66.7)/7 (58.3)	5 (33.3)/5 (41.7)	Fisher; p=0.65	1.43 (0.30-6.88)
IRR	+/-	6 (60)/11 (64.7)	4 (40)/6 (35.3)	Fisher; p=0.81	0.82 (0.16-4.09)

## Discussion

Vesicostomy is widely utilized to prevent upper urinary tract damage and control recurrent fUTIs in children with VUR, NB, or obstructive uropathy [1-4]. While this procedure effectively ensures urinary drainage and reduces bladder pressure, postoperative fUTIs are still observed in a considerable proportion of patients. In our study, 62.9% of patients developed fUTIs after vesicostomy, indicating that vesicostomy alone may not be sufficient to prevent infection in all cases.

The most significant finding of this study is the strong association between preoperative filling-phase VUR and postoperative fUTIs. Patients with filling-phase VUR demonstrated a markedly higher infection rate (75%) compared to those without (28.6%), with an OR of 7.50. This supports our hypothesis that low-pressure reflux during bladder filling contributes to bacterial ascent and infection risk even after vesicostomy creation [8-10]. These findings are consistent with previous reports indicating that reflux of contaminated urine during the filling phase can facilitate ascending infection, particularly in patients with persistently elevated intravesical pressures [11,12]. Consequently, patients with filling-phase VUR may benefit from closer monitoring and adjunctive measures, such as clean intermittent catheterization (CIC), as CIC has been shown to reduce infection rates and protect renal function in children with NB [13].

Other potential risk factors, including bladder compliance, DO, PUV, and IRR, did not show statistically significant associations with postoperative fUTIs. Although bladder dysfunction is known to predispose to urinary stasis and infection, our data suggest that bladder compliance and DO may not be reliable predictors of infection risk in this cohort. Inaccuracies and interobserver variability in pediatric UDS, especially in infants, may partly explain these results [13]. Indeed, the reproducibility of compliance and detrusor activity measurements in this age group remains limited due to physiological immaturity and difficulties in standardizing filling rates and voiding behavior [14].

A post hoc power analysis was conducted using the observed effect size between filling-phase VUR and the incidence of postoperative fUTIs [7]. Assuming an alpha level of 0.05 and observed proportions of 75% vs. 28.6%, the calculated statistical power with our sample size of 27 patients was approximately 0.78, suggesting that the study had adequate power to detect a clinically meaningful difference in infection outcomes. This post hoc power analysis (0.78) was considered appropriate for inclusion, as recommended during editorial review. Interestingly, while not statistically significant, younger age at surgery (<1 year) was associated with a higher rate of fUTIs (OR=1.5). This may be attributed to prolonged supine positioning in infants, which can facilitate retrograde urine flow and bacterial ascent. Although the observed power (0.78) was acceptable, larger cohorts would provide greater statistical robustness.

Our postoperative fUTI rate was higher than those reported by Prudente et al. (15%) and Rouzrokh et al. (38.1%) [2,15]. Notably, our patients had a lower median age at surgery (nine months), which may account for this discrepancy. Moreover, prior studies have emphasized that younger patients with severe preoperative bladder dysfunction derive significant benefit from temporary vesicostomy, improving upper tract morphology and renal function [3,15]. Additionally, Rouzrokh et al. reported that vesicostomy remains an effective, safe, and reversible procedure for children with refractory infections and hydronephrosis, particularly when performed early in the disease course [15].

The interpretation of UDS in infants and young children presents inherent challenges. Factors such as sedation, catheter size, patient cooperation, and bladder capacity variability can significantly influence measured parameters, limiting reproducibility and diagnostic precision. Furthermore, there is no universally accepted normative standard for urodynamic values in neonates and infants, complicating inter-institutional comparison and clinical interpretation [16]. These limitations underscore the importance of integrating radiographic and clinical findings, such as filling-phase VUR, into the preoperative evaluation rather than relying solely on UDS data to assess bladder risk.

This study has several limitations. First, due to the retrospective design and limited sample size, multivariate logistic regression could not be performed because of the limited number of events per variable, which may have affected the robustness of the findings. Given the limited events, per variable, multivariable logistic regression was not feasible and could risk overfitting; we therefore reported univariable estimates and prespecified this as a limitation. Second, although we clarified the definition of urinary tract infection (UTI) and exclusion of contaminated samples, the study did not analyze detailed urodynamic parameters such as maximum detrusor pressure (Pves), which may provide additional insight. Future prospective studies incorporating such parameters are warranted to validate our findings.

Despite its limitations, including retrospective design, single-institution setting, and small sample size, this study highlights the clinical utility of preoperative VCUG in identifying patients at elevated risk for postoperative infection. Prospective, multicenter studies with standardized UDS protocols are warranted to validate our findings and refine perioperative management strategies aimed at reducing infection recurrence.

In summary, by defining the diagnostic criteria for urinary tract infection, clarifying PUV evaluation methods, and acknowledging the limitations related to sample size and omitted urodynamic parameters, this study provides a reproducible and transparent framework for further investigation into postoperative infection risk in pediatric vesicostomy patients.

The findings of this study suggest that there appears to be an association between preoperative filling-phase urodynamic abnormalities and fUTI after vesicostomy; however, further large-scale studies are required to confirm this relationship.

## Conclusions

This study demonstrates that filling-phase VUR observed on preoperative VCUG represents a significant and independent risk factor for recurrent fUTIs following vesicostomy in pediatric patients. While vesicostomy effectively decompresses the bladder and protects the upper urinary tract, it may not be sufficient as a standalone intervention for children with filling-phase VUR. In such patients, the reflux of contaminated urine during the bladder filling phase likely persists even after diversion, maintaining a potential pathway for ascending infection.

Patients exhibiting filling-phase VUR should therefore be managed with heightened postoperative vigilance. This includes close monitoring for early signs of infection and consideration of adjunctive measures such as CIC and prophylactic antibiotic regimens, both of which have been shown to reduce infection recurrence and preserve renal function in children with neurogenic or dysfunctional bladders. Early identification of these high-risk patients through VCUG may facilitate individualized management strategies and improve outcomes. Furthermore, prospective multicenter studies are warranted to validate these findings and to establish evidence-based guidelines for postoperative care aimed at minimizing infection risk and preserving long-term renal function.
